# Phenotypes of Cornelia de Lange syndrome caused by non-cohesion genes: Novel variants and literature review

**DOI:** 10.3389/fped.2022.940294

**Published:** 2022-07-22

**Authors:** Huakun Shangguan, Ruimin Chen

**Affiliations:** Department of Endocrinology, Fuzhou Children’s Hospital of Fujian Medical University, Fuzhou, China

**Keywords:** Cornelia de Lange syndrome, non-cohesion, clinical diagnosis, whole exome sequencing, CdLS-like phenotypes

## Abstract

**Background:**

Cornelia de Lange syndrome (CdLS) is a genetic disorder caused by variants in cohesion genes including *NIPBL*, *SMC1A*, *SMC3*, *RAD21*, and *HDAC8.* According to the 2018 consensus statement, a patient with clinical scored ≥ 11 points could be diagnosed as CdLS. However, some variants in non-cohesion genes rather than cohesion genes can manifest as phenotypes of CdLS.

**Objectives:**

This study describes six variants of non-cohesion genes (*KDM6A*, *KMT2D, KMT2A ANKRD11*, and *UBE2A*), and assesses the reliability of 11-points scale criteria in the clinical diagnosis of CdLS.

**Methods:**

Whole-exome sequencing (WES) was performed on six patients with features of CdLS. Phenotypic and genotypic spectra of 40 previously reported patients with features of CdLS caused by non-cohesion genes variants and 34 previously reported patients with *NIPBL* variants were summarized. Clinical score comparison among patients with *NIPBL* variants versus those with variants in non-cohesin genes was performed.

**Results:**

Variants in non-cohesion genes were found in six patients [*KMT2A* (*n* = 2), *KMT2D*, *ANKRD11*, *KDM6A*, and *UBE2A*]. Of them, four variants (*KMT2A* c.7789C > T, *ANKRD11* c.1757_1776del, *KDM6A* c.655-1G > A, and *UBE2A* c.439C > T) were novel. Combining with previously reported cases, 46 patients with phenotypes of CdLS caused by variants in 20 non-cohesion genes are now reported. From this total cohort, the average clinical score of patients in *ANKRD11* cohort, *SETD5* cohort, and *AFF4* cohort was statistically lower than those in *NIPBL* cohort (8.92 ± 1.77 vs. 12.23 ± 2.58, 7.33 ± 2.52 vs. 12.23 ± 2.58, 5.33 ± 1.53 vs. 12.23 ± 2.58; *p* < 0.05). The average clinical score of *KMT2A* cohort, *EP300* cohort, and *NIPBL* cohort had not significantly different from (11 ± 2.19 vs. 12.23 ± 2.58, 10 ± 4.58 vs. 12.23 ± 2.58; *p* > 0.05).

**Conclusion:**

We described 4 novel variants of non-cohesion genes in six Chinese patients with phenotypes of CdLS. Of note, three genes (*KMT2D*, *KDM6A*, and *UBE2A*) causing features of CdLS have never been reported. The proposed clinical criteria for CdLS needed to be updated and refined, insofar as WES was necessary to confirm the diagnosis of CdLS. Our study expanded the spectra of non-cohesion genetic variations in patients with features of CdLS.

## Introduction

Cornelia de Lange syndrome (CdLS, OMIM # 122470, #614701, #610759, #300590, and #300882) is a rare genetic disorder caused by variants in cohesion complex genes including *NIPBL* (NM_133433.3), *SMC1A* (NM_006304.4), *SMC3* (NM_005445.3), *HDAC8* (NM_018486.2), and *RAD21* (NM_006265.3) (1). Up to 70% of CdLS patients are diagnosed with *NIPBL* variants. Around 5% of CdLS patients carry *SMC1A* variants, and 5% are *HDAC8* variants, less than 1% of CdLS patients are variants in *SMC3* or *RAD21* genes (2). The mode of inheritance of CdLS in the patient’s offspring could be either autosomal dominant (*NIPBL*, *RAD21*, or *SMC3* variants) or X-linked dominant (*SMC1*, or *HDAC8* variants) (2).

CdLS have variable phenotypes, such as microcephaly, minor facial dysmorphisms, intellectual disability, short stature, small hands, and hypertrichosis. In 2018, a diagnostic algorithm for initial evaluation of CdLS patients was proposed (3). Classic CdLS, based on clinical features, scored ≥ 11 points according to the consensus statement (3). Adopting this criteria would facilitate bedside diagnosis. However, some patients with features of CdLS were found to carry variants that were associated with other disorders, not with CdLS. Yuan et al. described a pathogenic *KMT2A* (NM_001197104.1) variant in one patient among 32 Turkish patients clinically diagnosed with classic CdLS (4). Cucco et al. also reported pathogenic variants in *EP300* (NM_001429.3) in a patient exhibiting resemblance to the classic CdLS phenotype in 2020 (5). With the development of contemporary genetic technology, it is not surprising that more patients with features of CdLS have been identified to carry variants in non-cohesion genes and, to date, the clinical genetic characteristics of these patients are ill-defined. Adding to the existing knowledge in this field is important in the quest to characterize the extent of heterogeneity of CdLS and Cornelia de Lange syndrome-like (CdLS-like) conditions.

In this study, we analyzed six patients with features of CdLS, and identified six variants in five non-cohesion genes. To date, 40 sporadic patients with features of CdLS caused by non-cohesion variants had been reported (4–17). We also evaluated the clinical features of these patients, and calculate the clinical scores of these patients according to the 2018 consensus statement to assess the reliability of 11-points scale criteria in the diagnosis of CdLS.

## Subjects and methods

### Subjects

All individuals in this study were evaluated by clinical geneticists and found to have clinical signs consistent with CdLS. Clinical details were retrospectively reviewed based on recent clinical criteria (3). This study was approved by the Ethics Committee of Fuzhou Children’s Hospital of Fujian Medical University, and written informed consents were obtained from the legal guardians of the patients.

### Whole-exome sequencing and variants interpretation

Genomic DNA was extracted from peripheral blood leukocytes of each patient. Blood samples from the parents were also collected. The Whole-exome sequencing (WES) was performed at Shanghai patient’s Medical Center. An adaptor-ligated library was prepared using SureSelect Human All Exon Kit (Agilent Technologies, Santa Clara, CA, America) according to the manufacturer’ s protocol. Target regions were sequenced on an Illumina Hiseq X Ten System (Illumina, San Diego, CA, America). Paired end reads were aligned to the GRCh37/hg19 human reference sequence. BAM files were generated by Picard and sequence variants were called by Genome Analysis Toolkit (GATK) Haplotype Caller. Variants were annotated by TGex and putative pathogenic variants detected in the patients by WES were validated by Sanger sequencing. Variants were classified following the American College of Medical Genetics and Genomics/Association for Molecular Pathology (ACMG/AMP) standards and guidelines (18).

### Literature review

We first searched the published literature in PUBMED, EMBASE and MEDLINE using the following search keys: (“Cornelia de Lange syndrome” OR “Cornelia de Lange syndrome-like” OR “CdLS” OR CdLS-like), without language restriction, with published data up to March 31, 2022. Then we reviewed the articles, and included the CdLS patients carrying variants in non-cohesion genes. We also used the following keys: (“Cornelia de Lange syndrome” OR “CdLS”) AND (“NIPBL”) to search the published literature in the same way. The *NIPBL* cases with detail clinical information were included.

### Statistics analysis

Statistical analysis for phenotypic among the patients with *NIPBL* variants versus those with variants in non-cohesion genes was performed by (corrected) the Chi-square test or Fisher’s exact test using GraphPad prism 8.0.1 software. In addition, the mean of clinical score within each group was analyzed using one way ANOVA test or Kruskal–Wallis test in GraphPad prism 8.0.1 software. *P* < 0.05 was considered statistically significant.

## Results

### Clinical phenotype of six patients in our study

The six patients exhibited overlapping phenotypes. The main characteristics were as following: facial dysmorphism (*n* = 6), intellectual disability or global developmental delay (*n* = 6), short stature (*n* = 6), small hands (*n* = 6), short 5th finger (*n* = 6), and microcephaly (*n* = 4). Detailed clinical information of the six patients are described in Table 1 and Supplementary Table 1.

**TABLE 1:** The genetic variants and clinical features of our six patients.

ID		P1	P2	P3	P4	P5	P6
Gender		Male	Female	Male	Male	Male	Male
Age (years)		5	0.83	3	1	2.5	0.58
Gene		*UBE2A*	*KMT2D*	*KDM6A*	*ANKRD11*	*KMT2A*	*KMT2A*
Variant		c.439C > T, p.Q147*	c.5845delC, p.Q1949Sfs*98	c.655-1G > A	c.1757_1776del, p.V586Efs*41	c.7789C > T, p.Q2597*	c.2629_2630delGA, p.D877fs*8
Inheritance		Materal	*De novo*	*De novo*	*De novo*	*De novo*	*De novo*
Novel or reported		Novel	Reported	Novel	Novel	Novel	Reported
ACMG classifcation		Pathogenic	Pathogenic	Pathogenic	Pathogenic	Pathogenic	Pathogenic
Main features (2 point each if present)	Synophrys and/or thick eyebrows	−	−	−	−	+	−
	Short nose, concave nasal ridge and/or upturned nasal tip	+	−	−	+	+	+
	Long and/or smooth philtrum	−	−	−	−	−	−
	Thin upper lip vermilion and/or downturned corners of mouth	−	+	+	−	+	+
	Hand oligodactyly and/or adactyly	−	−	−	−	−	−
	Congenital diaphragmatic hernia	−	−	−	−	−	−
Suggestive features (1 point each if present)	Small hands and/or feet	+	+	+	+	+	+
	Microcephaly	−	−	+	+	+	+
	Global developmental delay and/or intellectual disability	+	+	+	+	+	+
	Prenatal growth retardation	−	−	−	−	−	−
	Postnatal growth retardation	+	+	+	+	+	+
	Hypertrichosis	−	−	−	−	+	+
	Short fifth finger	+	+	+	+	+	+
Other features		With mouth, hypertelorism, high palate, inguinal hernia, renal cyst	Long palpebral fissures with eversion of the lower lid, ventricular septal defect, single transverse palmar crease, prominent fingertip pads	Long palpebral fissures with eversion of the lower lid, Long eyelashes, defect in the atrial septum, Coarctation of aorta, single transverse palmar crease	Macrodontia of the upper central incisors	Ptosis, long eyelashes, hypertelorism	Ptosis, long eyelashes, hypertelorism, cryptorchidism, patent ductus arteriosus
Clinical scored		6	6	7	7	12	10

### Molecular findings of six patients in our study

Whole-exome sequencing of the six individuals with clinically suspected CdLS identified six variants in *KMT2A*, *KMT2D* (NM_003482.3), *ANKRD11* (NM_013275.4), *KDM6A* (NM_021140.2), and *UBE2A* (NM_003336.2) (Table 1).

Patient #1 was a carrier of a materal nucleotide substitution in *UBE2A*: c.439C > T, resulting in a premature stop codon (p.Gln147*). Patient #2 showed a *de novo* nucleotide deletion in *KMT2D*: c.5845delC, resulting in a premature stop codon (p.Q1949Sfs*98). Patient #3 had a *de novo* 20 nucleotides deletion in *ANKRD11*: c.1757_1776del, resulting in premature stop codon (p.V586Efs*41). Patient #4 showed a *de novo* nucleotide substitution in *KMT2A*: c.7789C > T, leading to premature stop codon (p.Q2597*). Patient #5 had a *de novo* 2 nucleotide deletion in *KMT2A*: c.2629_2630delGA, resulting in a premature stop codon (p.D877fs*8). Patient #6 was a carrier of a *de novo* splice variant in *KDM6A*: c.655-1G > A. The SpliceTool^1^ predicted to delete 2 or 94 bp causing frameshift variants and premature termination, and multiple *in silico* tools predict deleterious outcomes of the splice variant (scored 1 in dbscsnv11_AdaBoost and 0.937 in dbscsnv11_RandomF orest). 4 variants (*UBE2A*: c.439C > T, *ANKRD11*: c.1757_1776del, *KMT2A*: c.7789C > T, and *KDM6A*: c.655-1G > A) were novel, and the *KMT2D* c.5845delC and *KMT2A* c.2629_2630delGA were both reported (19, 20). According to the ACMG/AMP standards and guidelines, these variants could all be classified as pathogenic (For *ANKRD11* c.1757_1776del, *KMT2A* c.7789C > T, and *KDM6A* c.655-1G > A: PVS1 + PS2 + PM2 + PP4; For *UBE2A* c.439C > T: PVS1 + PM2 + PP4; For *KMT2D* c.5845delC and *KMT2A* c.2629_2630delGA: PVS1 + PS2 + PP4).

### Phenotypes and genotypes of 46 patients caused by non-cohesion genes variants

To date, 40 patients with features of CdLS caused by variants in non-cohesion genes have been comprehensively described in the literature. Including the six patients in our study, a total of 46 pathogenic/likely pathogenic variants associated with 20 epigenetic genes including *ANKRD11* (*n* = 14), *BRD4* (NM_058243.3) (*n* = 2), *AFF4* (NM_014423.4) (*n* = 3), *KMT2A* (*n* = 6), *EP300* (*n* = 3), *SETD5* (NM_001080517.3) (*n* = 3), *ARID1B* (NM_001374820.1) (*n* = 2), *SMARCB1* (NM_003073.5) (*n* = 1), *TAF1* (NM_4606.5) (*n* = 1), *DDX23* (NM_004818.3) (*n* = 1), *CSNK1G1* (NM_022048.5) (*n* = 1), *ZMYND11* (NM_006624.7) (*n* = 1), *MED13L* (NM_015335.5) (*n* = 1), *PHIP* (NM_017934.7) (*n* = 1), *TAF6* (NM_005641.4) (*n* = 1), *NAA50* (NM_025146.4) (*n* = 1), *CREBBP* (NM_004380.3) (*n* = 1), *UBE2A* (*n* = 1), *KMT2D* (*n* = 1), and *KDM6A* (*n* = 1) are described (Supplementary Table 2). Of note, although we have reported the patient with *KMT2D* c.5845delC before, his phenotype consistent with CdLS was not recognized (19). Therefore, this case was not included in the literature review.

We divided these patients into six groups: *KMT2A* group, *ANKRD11* group, *EP300* group, *SETD5* group, *AFF4* group and a remaining group consisted of a lower number of patients with features of CdLS caused by non-cohesion genes variants (*BRD4*, *ARID1B, SMARCB1*, *TAF1*, *DDX23*, *CSNK1G1*, *ZMYND11*, *MED13L*, *PHIP*, *TAF6*, *NAA50*, *CREBBP*, *UBE2A*, *KMT2D*, and *KDM6A*). The average clinical score of patients in *KMT2A* group was 11 ± 2.19, 8.92 ± 1.77 in *ANKRD11* group, 10 ± 4.58 in *EP300* group, 7.33 ± 2.52 in *SETD5* group, 5.33 ± 1.53 in *AFF4* group and 8.88 ± 2.62 in the remaining group (Table 2).

**TABLE 2:** Clinical features of patients in CdLS-like cohort and those in *NIPBL* cohort.

Clinical feature	*NIPBL* cohort (*n* = 34)	*KMT2A* cohort (*n* = 6)	*ANKRD11* cohort (*n* = 14)	*EP300* cohort (*n* = 3)	*SETD5* cohort (*n* = 3)	*AFF4* cohort (*n* = 3)	Remaining genes cohort (*n* = 17)	Chi-square value	*P*-valve
Synophrys	31	5	10	3	2	3	14	5.049	0.45
Thick eyebrows	17	3	11	0	1	2	2^a^	17.772	0.002
Short nose	19	2	0^a^	1	0	1	3^a^	19.015	0.001
Concave nasal ridge	23	4	5	0^a^	2	0^a^	5^a^	14.383	0.011
Upturned nasal tip	22	2	11	2	1	1	7	8.204	0.192
Smooth philtrum	15	3	5	1	2	0	2	8.899	0.139
Long philtrum	28	3	13	1	1	1	10	14.287	0.012
Downturned corners of mouth	18	3	5	1	1	1	7	2.354	0.924
Thin upper lip vermilion	27	4	7	2	2	0	10	10.158	0.08
Adactyly	2	0	0	0	0	0	0	4.945	0.754
Hand oligodactyly	7	0	0	0	0	0	2	2.420	0.944
Small hands	17	3	8	0	0	0	6	8.008	0.205
Small feet	8	0	5	1	0	0	3	4.438	0.594
Microcephaly	24	5	7	2	0	1	11	8.558	0.164
Global developmental delay and/or intellectual disability	28	6	13	3	3	2	17	6.197	0.294
Prenatal growth retardation	23	1^a^	1^a^	1	0^a^	0^a^	4^a^	23.496	0.000
Postnatal growth retardation	23	6	1^a^	3	1	1	10	24.140	0.000
Hypertrichosis	13	4	2	3	0	0	2	16.143	0.005
Short fifth finger	12	5	6	1	0	0	7	8.137	0.194
Average clinical score (mean ± SD)	12.23 ± 2.58	11 ± 2.19^c^	8.92 ± 1.77^b^	10 ± 4.58	7.33 ± 2.52^b^	5.33 ± 1.53^bc^	8.88 ± 2.62^b^		< 0.0001

^a^Indicates that the incidence of phenotype was statistically significant compared with *NIPBL* cohort.

^b^Indicates that the average clinical score was statistically significant compared with *NIPBL* cohort.

^c^Indicates that the average clinical score of the two groups were statistically significant.

### Phenotypic and clinical score comparison of the non-cohesion cohort and *NIPBL* cohort

**FIGURE 1:**
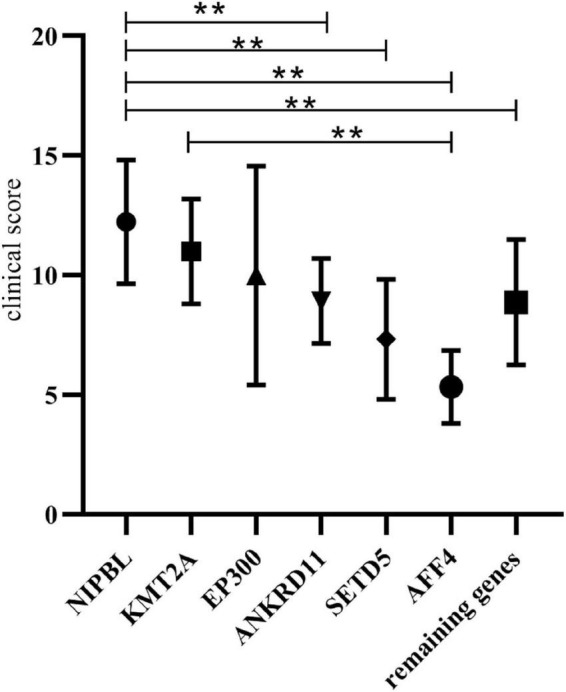
Clinical score comparison of the non-cohesion cohort and *NIPBL* cohort. **Indicates that the average clinical score of the two groups were statistically significant (*p* < 0.05).

We collected data on a group of 34 cases of CdLS caused by *NIPBL* variants from the literature and summarized the phenotypes and clinical score of the total 34 patients (clinical information and reference are shown in Supplementary Tables 3, 4). This affords us the opportunity to compare the clinical features and score of patients with *NIPBL* variants to those with variants in non-cohesion genes (Table 2 and Figure 1).

The overall clinical characteristics are similar among these groups, however, several differences still can be observed. The patients in *ANKRD11* cohort had lower frequencies of short nose, prenatal growth retardation and postnatal growth retardation than those in *NIPBL* cohort. The features of concave nasal ridge and prenatal growth retardation were more frequent in patients in *AFF4* cohort than those in *NIPBL* cohort. The features of concave nasal ridge and prenatal growth retardation showed significant statistical different from the patients in the *EP300* cohort and those in the *NIPBL* cohort. The frequencies of prenatal growth retardation in patients of *KMT2A* cohort showed lower than those of *NIPBL* cohort. Additionally, the average clinical score of patients in *ANKRD11* cohort, *SETD5* cohort and *AFF4* cohort was statistically lower than those in *NIPBL* cohort, respectively (8.92 ± 1.77 vs. 12.23 ± 2.58, 7.33 ± 2.52 vs. 12.23 ± 2.58, 5.33 ± 1.53 vs. 12.23 ± 2.58; *p* < 0.05). The average clinical score of patients in *KMT2A* cohort and *EP300* cohort both had no significantly difference with those in the *NIPBL* cohort (11 ± 2.19 vs. 12.23 ± 2.58, 10 ± 4.58 vs. 12.23 ± 2.58; *p* > 0.05).

## Discussion

In the present study, we provided our findings on the genetic analysis of six patients referred to our clinic for short stature. Short stature can be either a component of known syndromes or occur in undefined manifold complex clinical phenotypes. It was reported that 86% of CdLS presented with short stature, a common finding occurred in other rare genetic disorders, such as Wiedemann-Steiner syndrome (WDSTS, OMIM #605135) (75%), Kabuki syndrome (KS, OMIM #147920, #300867) (57%) and KBG syndrome (KBGS, OMIM #148050) (40–77%) (19, 21–23).

Several common clinical features were observed among the six patients, all of whom had short stature, moderate to severe development delay and/or intellectual disability, small hands and short fifth fingers, partly presenting with microcephaly. Hypertrichosis was also frequent. In addition, some CdLS-specific features including synophrys, concave nasal ridge and long smooth philtrum were observed, which in combination with short stature and intellectual disability resembled CdLS. However, the six patients with clinically suspected CdLS were found to carry 6 variants in *KMT2A*, *KMT2D*, *ANKRD11, KDM6A*, and *UBE2A*, which don’t function in cohesion protein. These identified variants in the genes (*KMT2A*, *KMT2D*, *KDM6A*, and *ANKRD11*) met the pathogenicity criteria according to ACMG/AMP standards and guidelines, including *de novo* occurrence, absence in general population and predicted LOF effect causing diseases through this pathogenetic mechanism. The *UBE2A* c.439C > T variant was also deemed pathogenic given that the variant causes a premature stop codon and is absent in the general population.

*KMT2A* variants are associated to the WDSTS, a rare autosomal dominant condition characterized by different debilities, mainly intellectual disability, short stature, hypertrichosis, distinctive facial features (thick eyebrows, long eyelashes, narrow palpebral fissures, hypertelorism, ptosis, broad nasal tip), and skeletal abnormalities (clinodactyly, brachydactyly, advanced bone age) (24). Two patients (P5 and P6) carrying *KMT2A* variants in our study shared a number of clinical features of CdLS including short nose, anteverted nares, concave nasal ridge, short stature, global developmental delay, small hands, short 5th finger and microcephaly. And both had clinical scores of 12 and 10, respectively (Table 1). Previously, four patients with features of CdLS found to carry *KMT2A* variants have been reported. With our new two patients, the average clinical score of the six patients was no significantly different from a cohort of *NIPBL* patients (11 ± 2.19 vs. 12.23 ± 2.58), which infers that some patients with *KMT2A* variants may be misdiagnosed as CdLS without molecular test. However, the combination of ptosis and long eyelashes was more frequent in *KMT2A* patients than *NIPBL* patients (24). Four of these six patients (two patients in our study and two patients in literature) had features of ptosis and hypertelorism, which may help to distinguish WDSTS and CdLS (13, 16).

KBG syndrome is a rare condition characterized by intellectual disability, global developmental delay, short stature, skeletal anomalies, distinctive facial features, and macrodontia of the upper central incisors (23). One child (P4) in our study had a frameshift variant in *ANKRD11* gene. He presented with some features of CdLS including anteverted nares, short stature, global developmental delay, microcephaly, small hands and short 5th finger (Table 1; scored 7). He also presented with macrodontia of the upper central incisors, which is a component of KBGS (25). Although KBG has clinical features overlapping with CdLS, the average clinical score is lower than the *NIPBL* cohort. Additionally, the features of short nose, prenatal and postnatal growth retardation had a lower frequency in *ANKRD11* cohort than those in the *NIPBL* cohort (Table 2). More importantly, a specific combination of a triangular shaped face and a bulbous nasal tip was crucial for the accurate clinical diagnosis of KBGS and CdLS (17).

Additionally, we reported a novel patient (P1) with nonsense variant in *UBE2A*, involved in coding ubiquitin-conjugating enzyme E2A, with CdLS related phenotypes. Haploinsufficiency of *UBE2A* underlies the X-linked intellectual disability type Nascimento (XIDTN, OMIM #300860), also known as UBE2A deficiency syndrome (26). To date, about 40 patients with XIDTN had been reported in literature (26). Nascimento et al. reported that its distinct abnormalities including a myxoedematous appearance and nail dystrophies, yet these features were not observed in P1. Additionally, prominent supraorbital ridge, hypertelorism, and prominent columella/hypoplastic alae nasi and a wide mouth were also common in XIDTN (27). However, except a wide mouth, these features were not found in our patient. Interestingly, the phenotypic features of our patient with *UBE2A* variant shared similarities with the previously described CdLS individuals including anteverted nares, short stature, intellectual disability, small hands and short 5th finger (Table 1, scored 6). These findings suggested that individuals with *UBE2A* variant instead present with specific features that is only minimally overlapping with CdLS.

Our two patients (P2 and P3) found to carry *KTM2D* and *KDM6A* variants, presented with thin upper lip vermilion, downturned corners of mouth, a finding mainly described in CdLS (Table 1; score 6 and 7, respectively) (3). Pathogenic variants in the chromatin regulatory methyltransferase gene *KMT2D* and demethyltransferase gene *KDM6A* cause autosomal dominant KS, associated with specific clinical signs including arched eyebrows, long palpebral fissures with eversion of the lower lid, large protuberant ears, and prominent fingertip pads, hypoplastic left heart (28). Corresponding to it, the two patients both had long palpebral fissures with eversion of the lower lid and hypoplastic left heart, features that help to distinguish KS and CdLS. Additionally, P2 had prominent fingertip pads which are considered a distinctive of KS.

A total of 46 patients with feature of CdLS caused by variants in non-cohesion genes were summarized. We collected data on a group of 34 cases of CdLS caused by *NIPBL* variants from the literature and summarized the phenotypes and clinical score of those patients. This affords us the opportunity to compare the clinical features and score of patients with *NIPBL* variants to those with variants in non-cohesion genes. Our results suggested that *KMT2A* and *EP300* can be included within the extended list of CdLS genes that are studied in CdLS panels. In addition, the average clinical score of *ANKRD11* cohort, *SETD5* cohort, and *AFF4* cohort was 8.92 ± 1.77, 7.33 ± 2.52, 5.33 ± 1.53, respectively. Variants in these three genes caused limited phenotypes overlapping with CdLS. Single case with variants in some non-genes causes features of CdLS were reported in literature. The presence of one or two cases made statistical analysis impossible. Additional case accumulation is needed to further explore the relationship between these non-genetic variants and phenotypes of CdLS.

Extensive evidences showed that the cohesion complex functions in sister chromatid cohesion, as well as playing a role in the regulation of transcription. Some reports suggested that the cohesion genes including *NIPBL*, *SMC1A*, *SMC3*, *RAD21*, and *HDAC8* (29–31), are involved in chromatin-mediated transcriptional regulation. Moreover, function experiments showed cell lines of individuals with CdLS displayed global transcriptional disturbances rather than cohesion defects (32). Additionally, the cohesion components and chromatin-remodeling proteins strongly interact (33). These studies provided a new perspective on the distinct roles of epigenetic mechanism of phenotypes of CdLS. As a consequence, identifying the causes of patients with features of CdLS by WES is a requisite for accurate genetic diagnosis.

### Conclusion

We describe four novel variants in six Chinese patients with features of CdLS caused by variants in four non-cohesion genes (*ANKRD11*, *KMT2D*, *KDM6A*, and *UBE2A*). In addition, three genes (*KMT2D*, *KDM6A*, and *UBE2A*) causing phenotypes of CdLS have never been reported. Some patients carrying variants in non-cohesion genes could be misdiagnosed as CdLS solely based on the 11-points scale criteria, and WES was necessary to confirm the diagnosis of CdLS. This study expands the spectra of non-cohesion genetic variations in patients with features of CdLS.

## Data availability statement

The original contributions presented in this study are included in the article/Supplementary Material, further inquiries can be directed to the corresponding author.

## Ethics statement

The studies involving human participants were reviewed and approved by the Ethics Committee of Fuzhou Children’s Hospital of Fujian Medical University. Written informed consent to participate in this study was provided by the participants’ legal guardian/next of kin.

## Author contributions

HS conducted the data analysis and interpretation and wrote the manuscript. RC contributed to the study design, helped to analyze data, and revise the first draft. Both authors contributed to the article and approved the submitted version.

## Conflict of interest

The authors declare that the research was conducted in the absence of any commercial or financial relationships that could be construed as a potential conflict of interest.

## Publisher’s note

All claims expressed in this article are solely those of the authors and do not necessarily represent those of their affiliated organizations, or those of the publisher, the editors and the reviewers. Any product that may be evaluated in this article, or claim that may be made by its manufacturer, is not guaranteed or endorsed by the publisher.

## Footnotes

1
https://rddc.tsinghua-gd.org/tools/split-tool/result?uuid=f8b645a7-9dab-4683-bcc4-00d00711873f

## Abbreviations

CdLS: Cornelia de Lange syndrome
RAD21: RAD21 cohesin complex component
NIPBL: Nipped-B-like protein
SMC1A: Structural Maintenance of Chromosomes 1A
HDAC8: Histone deacetylases
SMC3: Structural Maintenance of Chromosomes 3
KS: Kabuki syndrome
KMT2D: lysine (K)-specific methyltransferase 2D
KDM6A: lysine (K)-specific methylase 6A
UBE2A: ubiquitin-conjugase E2A
ANKRD11: Ankyrin Repeat Domain 11
KMT2A: lysine (K)-specific methyltransferase 2A
WES: whole-exome sequencing
ACMG/AMP: American College of Medical Genetics and Genomics and the Association for Molecular Pathology
BRD4: Bromodomain containing 4
AFF4, AF4/FMR2 family: member 4
EP300: E1A binding protein p300
SETD5: SET domain containing 5
SMARCB1: SWI/SNF related, matrix associated, actin dependent regulator of chromatin subfamily b, member 1
TAF1, TAF1 RNA polymerase II: TATA box binding protein (TBP)-associated factor
DDX23: DEAD-box helicase 23
CSNK1G1, casein kinase 1: gamma 1
ZMYND11, zinc finger: MYND-type containing 11
MED13L: Mediator complex subunit 13-like
PHIP: pleckstrin homology domain interacting protein
TAF6, TAF6 RNA polymerase II: TATA box binding protein (TBP)-associated factor
NAA50: N-Terminal Acetyltransferase 50
CREBBP: CREB binding protein.
